# Urinary Analysis and Diagnosis by Microscopical and Chemical Examination

**Published:** 1906-09

**Authors:** 


### BOOKS AND AUTHORS.

**Urinary Analysis and Diagnosis by Microscopical and Chemical Examination.** By **Louis Hertzmann,** M.D., of New York. Second edition, revised and enlarged, with 131 illustrations. Octavo pp. 319. New York: William Wood & Company. 1906. (Price: $2.00, Cloth).

With budding urologists and full-blown analysts rather overfilling the field of urologic literature, it is rather more than a mere pleasure to have so able a work as Hertzmann's brought forth in a second edition. This book is nothing in the nature of a miracle worker. It has none of the egotistic ear marks of superior being and it doesn't pretend to make over either a student or a practitioner of medicine into a glittering expert, after a casual reading. It is, on the contrary, a quiet, sensible, well-balanced exposition of a field which has been receiving scant attention up to the present time—namely, microscopic examination and microscopic diagnosis. Complicated tests have no place in the book; but the everyday working tests in the analysis of urine, tests which tell, are explained in detail, and this in itself makes it a really valuable working volume as well as a very helpful one.

It is in the microscopic department of examination and diagnosis that the real worth of the book shows forth. Great stress is laid on these departments, which is proper, because, as every man who works seriously and systematically in the genitourinary field knows, there are many cases in which clinical symptoms, though pointing to the genitourinary tract, are vague. In such cases even a careful and painstaking analysis of urine fails to give a positive finding, yet often the microscope clears away the mists of doubt and points the way to correct diagnosis and rational treatment. A feature which adds to the value of the book is the illustrations, the majority of which were drawn by the author from his own clinic, hence are correct. Their usefulness is increased by their size, nearly all being full page plates in which every detail of microscopic finding is brought out clearly.

Divided into three parts the book covers the entire field of its usefulness in an admirable manner. The first part deals with the chemic analysis of urine; the second with microscopic examination; and the third with microscopic diagnosis. Added to these are pages devoted to diseases of the male and female sexual organs, the determination of the functional efficiency of the kidney, to histology, and to secretion, all of which are unusually lucid. The general remarks on the methods of study and examination, and the advice of the author in matters which might naturally arise in so important a subject, are excellent and clear up the doubts which often becloud the view of a new and ill-understood subject. Too much credit cannot be accorded Hertzmann for the excellence of the drawings he has furnished and the publishers are to be congratulated on having accepted the pen and ink sketches of microscopic findings, reproducing them in full page plates. It indicates that they prefer scientific certainty to prettily printed pages of indistinct half pages. A careful study of the book will make genitourinary diagnosis easier and lead to more sensible treatment,—for all of which the patient ought to feel profounding thankful. N. W. W.

